# Supplementary material for: Gender differences in suicide-related communication of young suicide victims
Source: PLoS One. 2021 May 21;16(5):e0252028. doi: 10.1371/journal.pone.0252028 (PMC8139476; doi:10.1371/journal.pone.0252028)
Supplement: S2 File — (DOCX) [file pone.0252028.s002.docx]

# **Coding system for qualitative analyses**

The following coding system has been used to analyze suicide-related communication in youths under 20 who died by suicide in the Netherlands in 2017. In this article, we adhere to Frey et al.’s(1) definition of personal suicide-related communication (SRC) for our analysis, which is *“the act of conveying one’s own suicide ideation, intent, or behaviours to another person”.* A suicide-related communication event is defined in this study as a moment in the life of the youth, as described by the respondent, in which SRC occurred.

*Explicit* SRC events have been analyzed throughout the life course. These are SRC events that clearly reflected suicide ideation or intent of the youth, i.e. a suicide attempt or open disclosure of suicidal thoughts, or a preoccupation with death or dying that was notable to next-of-kin (NOK). Conversely, *implicit* events were analyzed only when they occurred in the last months. This was based on the assumption that suicide ideation had manifested by that time in the majority of youths.

| Throughout life course | Last months |
| --- | --- |
| Explicit SRC Verbal/nonverbal/written SRC | Explicit and implicit SRC Verbal/nonverbal/written SRC |

The codes ‘explicit’ and ‘implicit’ were consistently coded in pairing with a code for ‘verbal’, ‘nonverbal’ or ‘written’. Thus, every event was one of 6 combinations. These combinations are outlined below.

### **Explicit/verbal**

A verbal expression of the youth is described by respondents, which clearly reflects that the youth has suicide ideation or intent. Some examples are: *“I don’t like living anymore”*, or *“I wish I was dead*”, or “*I think about what it would be like if I did not exist anymore*”.

### **Explicit/nonverbal**

The respondent explains a specific behaviour of the youth that clearly suggests suicide ideation or intent in the youth. This entails, for example, preparing for a suicide, or attempting a suicide. Note that in this study, we included deliberate self-harm in this code combination, as an explicit nonverbal expression of suicidality.

DSH is the only explicit nonverbal event we have coded that is not unequivocally associated with suicide ideation. Two reasons underly this decision: [1] NOK in our sample consistently framed deliberate self-harm as a signal that their child had suicidal thoughts and [2] several researchers have argued that deliberate self-harm should be conceptualized in relation to suicide regardless of clear intention, because of the high co-occurrence between deliberate self-harm and suicide attempt(2,3). This would suggest that deliberate self-harm is associated with an intention higher than 0 to not want to live anymore. Lastly, we believe that deliberate self-harm, regardless of suicidal intent, reflects one’s depreciation of physical integrity and contributes to an acquired capability for suicide, which is a key risk factor to die by suicide(4).

### **Explicit/written**

Respondents provide information about a written communication by the hand of the youth, which clearly shows that the youth had suicide ideation or intent. These include similar statements as noted under explicit verbal, such as: *“I think about ending my life”.*

### **Implicit/verbal**

Interview data in which the respondent describes a verbal expression of the youth in the last months of their life that has no clear link to suicide, dying or death, but which respondents associated with suicide ideation or intent in the youth. These could be a wide variety of verbal expressions, such as: *“I am not going to be old anyway”*, or communication of distress such as: *“my thoughts are really driving me crazy”*.

### **Implicit/nonverbal**

Implicit nonverbal events entail the presentation of a notable behaviour of the youth in the last months of their life which respondents perceived as a signal of suicide ideation or intent of the youth, but which does not have a clear link to suicide, dying or death. Example behaviour can be changing sleeping patterns or withdrawal from social activities.

### **Implicit/written**

Respondents describe how the youth wrote something in the last months which does not directly relate to suicide, dying or death but which, according to them, suggests that suicide ideation or intent was present in the youth. These can for example be diary entries that report on sad or anxious feelings.

# **Code list**

Codes other than the combinations of SRC_explicit/implicit and SRC_form, have been used situationally. Below is the complete list of all codes used in this study.

| **Code** | **Operational definition** |
| --- | --- |
| SRC_explicit | The respondent describes how the youth explicitly gave notice of his or her suicide ideation or intent. This entails any expression (verbal, nonverbal, or written) that has a direct relation to suicide, death, dying, or the end of existence. It includes various events, depending on the form (see above). |
| SRC_implicit | The respondent describes how the youth implicitly communicated suicide ideation or intent. This entails any expression (verbal, nonverbal or written) that does not have a direct relation to suicide, death, dying or the end of existence, but which according to the respondent was a signal that suicide ideation or intent was present. Thus, central in implicit SRC events is the perception of NOK. |
| SRC_form_verbal | An SRC event in which the youth expressed in words his or her suicide ideation or intent, implicitly or explicitly, to one or more other individuals. |
| SRC_form_nonverbal | An SRC event in which the youth expressed his or her suicide ideation or intent, implicitly or explicitly, by means of actions, interests or behaviours other than verbal expressions or written text. |
| SRC_form_written | An SRC events in which the youth expressed his or her suicide ideation or intent, implicitly or explicitly, in written text. |
| SRC_content | This code is only applicable to verbal or written SRC. It is a fragment of interview data in which the respondent describes, paraphrases, or quotes the content of a verbal or written SRC event. |
| SRC_medium | A medium other than face-to-face communication was used in an SRC event as described by the respondent. |
| SRC_medium_phone | The SRC event entailed a communication that was transmitted by phone |
| SRC_medium_messenger | The SRC event entailed a communication that was formulated in a text message or messenger app |
| SRC_medium_social media and forums | The SRC event entailed a communication that was shared through social media or an online forum |
| SRC_medium_letter | The SRC event entailed a communication that was sent in a letter |
| SRC_medium_diary/notes | The SRC event entails a diary entry or personal notes that have been found during or after the life of the youth. Note that we have not distinguished between these, as the data was rarely conclusive on the time of finding the communication. |
| SRC_recipient | The respondent mentions a recipient other than themselves in an SRC event |
| SRC_interpretation | The respondent describes his or her interpretation or perception of an SRC event |
| SRC_response | The respondent describes his or her response to a specific SRC event. This can be a verbal response, but also an action such as help-seeking. |
| SRC_communication_barrier | The respondent explains individual challenges to understand a communication event of the youth, or highlights barriers for them to talk openly about suicide with the youth. |
| SRC_debut | The SRC event that, based on the interview data, was the first SRC event to occur in the life of the youth. |
| SRC_last_months | A code used for any SRC event that took place in the last months alive of the youth. |
| SRC_last_event | The SRC event that, based on the interview data, was the SRC event to occur temporally closest to the suicide act. |

## **References**

1. Frey LM, Fulginiti A, Sheehan L, Oexle N, Stage DL, Stohlmann-Rainey J. What’s in a word? Clarifying terminology on suicide-related communication. Death Stud. 2020;44(12):808–18.

2. Andover MS, Morris BW, Wren A, Bruzzese ME. The co-occurrence of non-suicidal self-injury and attempted suicide among adolescents: distinguishing risk factors and psychosocial correlates. Child Adolesc Psychiatry Ment Health. 2012 Mar;6:11.

3. Kapur N, Cooper J, O’Connor R, Hawton K. Non-suicidal self-injury v. attempted suicide: new diagnosis or false dichotomy? Br J Psychiatry. 2013;202 5:326–8.

4. Joiner T. Why People Die by Suicide [Internet]. Vol. 1st Harvar. Cambridge, Mass: Harvard University Press; 2007. Available from: https://search.ebscohost.com/login.aspx?direct=true&db=e000xww&AN=282447&site=ehost-live&scope=site
