# Supplementary material for: Gender differences in suicide-related communication of young suicide victims
Source: PLoS One. 2021 May 21;16(5):e0252028. doi: 10.1371/journal.pone.0252028 (PMC8139476; doi:10.1371/journal.pone.0252028)
Supplement: S3 File — (DOCX) [file pone.0252028.s003.docx]

**Consolidated criteria for reporting qualitative studies (COREQ): 32-item checklist**

Tong A, Sainsbury P, Craig J. Consolidated criteria for reporting qualitative research (COREQ): a 32-item checklist for interviews and focus groups. International journal for quality in health care. 2007 Dec 1;19(6):349-57.

**Domain 1: Research team and reflexivity**

Personal Characteristics

1. Interviewer/facilitator Which author/s conducted the interview or focus group?

The interviews were conducted by authors Elias Balt, Milou Looijmans, Diana van Bergen, and three senior interviewers who are not among the authors but are credited for their efforts in the acknowledgements of the Mérelle and colleagues’ 2020 article.

1. Credentials What were the researcher’s credentials? E.g. PhD, MD

The authors’ credentials and affiliations are mentioned under the ‘about the authors’ tab and on the title page of the manuscript.

1. Occupation What was their occupation at the time of the study

Elias Balt: Junior Researcher (currently PhD student)

Saskia Mérelle: Senior researcher

Diana Van Bergen: Associate professor of Education and senior researcher

Renske Gilissen: Manager at a Research Department

Pommeline van der Post: Research intern

Milou Looijmans: Junior Researcher (currently PhD Student)

Daan Creemers: Clinical Psychologist and Research Coordinator

Sanne Rasing: Senior researcher

Wico Mulder: Youth doctor

Lieke van Domburgh: Manager of a Residential Youth Care Unit

Arne Popma: Professor of Child and Adolescent Psychiatry and Child and Adolescent Psychiatrist

1. Gender Was the researcher male or female?

The research team and interview team consisted of slightly more persons with a female identity than a male identity (60/40)

1. Experience and training What experience or training did the researcher have?

The researchers were trained as academics and/or mental health care professionals, and their background is in the behavioural sciences, health sciences, social sciences, and medical sciences.

Relationship with participants

1. Relationship established. Was a relationship established prior to study commencement?

In one case, parents who were part of the sample were also part of a series of test interviews. They were interviewed twice (the first time in the testing phase of the instrument, and the second time as part of the study, yet by different interviewers). In all other cases, the informants had never heard of the study nor had they met the researcher prior to taking part in the study.

1. Participant knowledge of the interviewer

What did the participants know about the researcher? e.g. personal goals, reasons for doing the research.

The participants received an information letter that, amongst other aspects, explained who the research team and research institute were that conducted the study. The information letter was approved by the Medical Research Ethics Committee (MREC) of Amsterdam UMC.

1. Interviewer characteristics What characteristics were reported about the interviewer/facilitator? e.g. Bias, assumptions, reasons and interests in the research topic

We assume that the variety in gender, age, sexual orientation as well as the multidisciplinary research team and advisory board has helped us to overcome potential biases, as we held a series of reflection meetings during all phases of the research project.

**Domain 2: study design**

The data for the current research has been obtained from an earlier psychological autopsy study, by Mérelle et al (2020), titled: *“A multi-method psychological autopsy study on youth suicides in the Netherlands in 2017: Feasibility, main outcomes, and recommendations”*. Therefore, the answers to most questions concerning the participant selection and the data collection are answered in the methods section of the original article of Mérelle et al. (2020).

Other questions which require specific answers in accordance with the current manuscript will be answered.

Theoretical framework

1. Methodological orientation and Theory: What methodological orientation was stated to underpin the study? e.g. grounded theory, discourse analysis, ethnography, phenomenology, content analysis

The Constant Comparative Method has been employed in the current study (see methodology of the current manuscript, P. 7)

Participant selection

1. Sampling How were participants selected? e.g. purposive, convenience, consecutive, snowball

The participant selection is detailed in the article of Mérelle et al. (2020), on pages 7-8.

1. Method of approach How were participants approached? e.g. face-to-face, telephone, mail, email

The participant selection is detailed in the article of Mérelle et al. (2020), on pages 7-8.

1. Sample size How many participants were in the study?

The participant selection is detailed in the article of Mérelle et al. (2020), on pages 11-14.

1. Non-participation How many people refused to participate or dropped out? Reasons?

The participant selection is detailed in the article of Mérelle et al. (2020), on pages 11, 14-15.

Setting

1. Setting of data collection Where was the data collected? e.g. home, clinic, workplace

The setting of the research is detailed in the article of Mérelle et al. (2020), on page 8.

1. Presence of non-participants Was anyone else present besides the participants and researchers?

The setting of the research is detailed in the article of Mérelle et al. (2020), on page 8.

1. Description of sample What are the important characteristics of the sample? e.g. demographic data, date

An extensive demographic profile of the sample is detailed in the article of Mérelle et al. (2020), on pages 11-14. Basic demographics deemed relevant to answer the research questions of the current study have been described in the current manuscript on page 8.

Data collection

1. Interview guide. Were questions, prompts, guides provided by the authors? Was it pilot tested?

A description of the interview process is detailed in the article of Mérelle et al, on pages 8-11.

1. Repeat interviews. Were repeat interviews carried out? If yes, how many?

A description of the interview process is detailed in the article of Mérelle et al, on pages 8-11.

1. Audio/visual recording. Did the research use audio or visual recording to collect the data?

A description of the interview process is detailed in the article of Mérelle et al, on pages 8-11.

1. Field notes Were field notes made during and/or after the interview or focus group?

A description of the interview process is detailed in the article of Mérelle et al, on pages 8-11.

1. Duration. What was the duration of the interviews or focus group?

A description of the interview process is detailed in the article of Mérelle et al, on pages 8-11.

1. Data saturation. Was data saturation discussed?

Data saturation concerning all major elements of the original psychological autopsy study has been discussed at several times during the data collection process.

1. Transcripts returned. Were transcripts returned to participants for comment and/or correction?

All informants were allowed to see the verbatim transcript of their interview, but not allowed to comment or correct the transcript.

**Domain 3: analysis and findings**

Data analysis

1. Number of data coders How many data coders coded the data?

The data was sequentially coded by two researchers (EB, PP). and the applied codes were reviewed by a third researcher (SM).

1. Description of the coding tree Did authors provide a description of the coding tree?

The coding tree and coding approach has been made available in the supporting information of the current manuscript.

1. Derivation of themes. Were themes identified in advance or derived from the data?

Central themes were identified a priori to data analyses, as guided by the research question. Several subordinate themes have emerged from the data. See page 6-7 of the current manuscript (methodology).

1. Software What software, if applicable, was used to manage the data?

An ATLAS.ti version 8.3 software package was used to manage and code the data.

1. Participant checking Did participants provide feedback on the findings?

Participants were provided with the opportunity to read the original research report of Mérelle et al. (2020), in Dutch, before publication. Two participants provided feedback to the main findings, which were accordingly incorporated and adjusted. This feedback has been considered for the present manuscript when there was a relation to SRC, but no separate member check has been organized.

Reporting

1. Quotations presented. Were participant quotations presented to illustrate the themes / findings? Was each quotation identified? e.g. participant number

The quotations were selected to illustrate the findings. The respondent type (e.g. parent, peer) of individual quotes has been noted in the manuscript. Any elements of the quotes that might have jeopardized the anonymity of the participants have been removed or adjusted (e.g. dates, names, unique details).

1. Data and findings consistent. Was there consistency between the data presented and the findings?

See result section on pages 8-16 of the manuscript.

1. Clarity of major themes. Were major themes clearly presented in the findings?

In our manuscript we aimed to be complete and accurate in presenting important themes in the suicide-related communication of youths. The different patterns identified for boys and girls are presented and illustrated by means of case examples and/or respondent quotes. To provide an indication of the weight of specific patterns, we provide information about the number of young persons to whom a major theme was applicable.

1. Clarity of minor themes. Is there a description of diverse cases or discussion of minor themes?

Unique findings, as well as divergence from the identified major gender patterns in suicide-related communications, are specifically mentioned. These findings are supported by examples or respondent quotes only if we believed this would add to the comprehensiveness of the results, in order to be more concise in the manuscript.
